# Unveiling the molecular basis of paracetamol-induced hepatotoxicity: Interaction of *N*-acetyl-*p*-benzoquinone imine with mitochondrial succinate dehydrogenase

**DOI:** 10.1016/j.bbrep.2024.101727

**Published:** 2024-05-07

**Authors:** Md Sahadot Hossen, Adiba Akter, Mahir Azmal, Mostakim Rayhan, Kazi Saiful Islam, Md Mahmodul Islam, Shamim Ahmed, Mohammad Abdullah-Al-Shoeb

**Affiliations:** a Department of Biochemistry and Molecular Biology, Shahjalal University of Science and Technology, Sylhet 3114, Bangladesh; b Department of Biochemistry and Molecular Biology, Jahangirnagar University, Savar, Dhaka, Bangladesh; c Department of Pharmacy, Noakhali Science and Technology University, Noakhali 3814, Bangladesh

**Keywords:** Acetaminophen, KEGG pathway, Molecular docking analysis, Molecular dynamics simulation, NAPQI, Paracetamol toxicity

## Abstract

**Background and aim:**

*N*-acetyl-*p*-benzoquinoneimine (NAPQI), a toxic byproduct of paracetamol (Acetaminophen, APAP), can accumulate and cause liver damage by depleting glutathione and forming protein adducts in the mitochondria. These adducts disrupt the respiratory chain, increasing superoxide production and reducing ATP. The goal of this study was to provide computational proof that succinate dehydrogenase (SDH), a subunit of complex II in the mitochondrial respiratory chain, is a favorable binding partner for NAPQI in this regard.

**Method:**

Molecular docking, molecular dynamics simulation, protein-protein interaction networks (PPI), and KEGG metabolic pathway analysis were employed to identify binding characteristics, interaction partners, and their associations with metabolic pathways. A lipid membrane was added to the experimental apparatus to mimic the natural cellular environment of SDH. This modification made it possible to develop a context for investigating the role and interactions of SDH within a cellular ecosystem that was more realistic and biologically relevant.

**Result:**

The molecular binding affinity score for APAP and NAPQI with SDH was predicted −6.5 and −6.7 kcal/mol, respectively. Furthermore, RMSD, RMSF, and Rog from the molecular dynamics simulations study revealed that NAPQI has slightly higher stability and compactness compared to APAP at 100 ns timeframe with mitochondrial SDH.

**Conclusion:**

This study serves to predict the mechanistic process of paracetamol toxicity by using different computational approaches. In addition, this study will provide information about the drug target against APAP hepatotoxicity.

## Highlights

• Higher predicted binding affinity of Succinate dehydrogenase with NAPQI (−6.7 kcal/mol) compared to APAP (−6.5 kcal/mol).
• NAPQI and APAP bind to the FAD binding site, occupying the LysA50 and AlaA179 residues in SDH flavin subunit.
• Both NAPQI and APAP exhibit compact and rigid binding with the FAD site of SDH in 100 ns of Molecular dynamics simulation.
• The favorable binding of NAPQI and APAP with SDH may represent mechanistic pathway for APAP-induced liver injury.

## Graphical abstract

**Figure:**
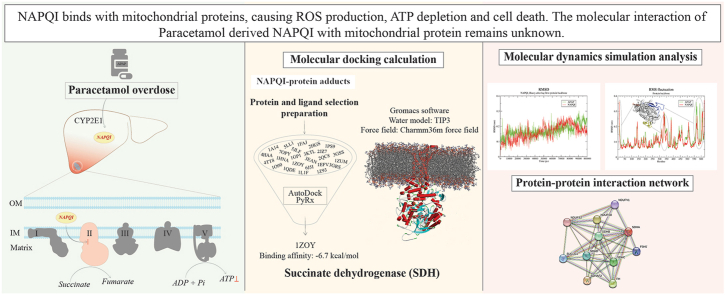

## 1 Introduction

Acetaminophen, commonly referred to as paracetamol or APAP, is widely utilized for its non-narcotic pain-relieving and fever-reducing qualities [1,2]. Currently, paracetamol also used as combination drug with opioids for severe cancer pain [3]. Although typically safe when used at recommended therapeutic doses, the excessive ingestion of this over-the-counter drug can potentially cause hepatotoxicity, which may lead to acute liver failure (ALF) [4,5]. Along with ALF, acetaminophen overdose also associated with acute kidney injury, gastrointestinal ulceration, bronchospasm, and ductus arteriosus, etc. [3]. The majority of ALF cases in both the United States and Great Britain are primarily attributed to APAP-induced hepatotoxicity [[6], [7], [8], [9]].

Metabolism of APAP occurs in liver microsomes in three phases: major quantities (90 %) are detoxified by glucuronidation and sulfation processes, thereby turning into nontoxic metabolites; small amounts (2 %) are excreted through urine without further processing; and only 5–9% of ingested paracetamol undergo direct terminal oxidations by cytochrome P-450 (CYP2E1)-into highly toxic and reactive metabolites *N*-acetyl-*p*-benzoquinonimine (NAPQI) [[10], [11], [12]]. This NAPQI is very unstable toxic intermediate undergo quick detoxification by glutathione conjugation [12,13]. Due to intentional or unintentional over dosage of APAP causes the excessive production of NAPQI and subsequently depleted the glutathione level. Only approved therapeutic for APAP-induced liver injury is N-Acetylcysteine (NAC), which can replenish the glutathione level if treated immediately after APAP over dosage [14]. If untreated, excessive NAPQI can bound with mitochondrial intermembrane proteins or cellular macromolecules, though the precise process is still up for debate [10,[15], [16], [17]].

In APAP-induced hepatotoxicity, mitochondria play a crucial role. Interactions with mitochondrial proteins and the resulting production of oxidative stress are involved in the early stages of hepatic injury [18]. The propagation or amplification phases come next, and they result in hepatocellular necroptosis [16,[18], [19], [20], [21]]. In addition, the reactive oxidative metabolite NAPQI reduced succinate-driven ATP generation and specifically inhibited mitochondrial complex II in mouse model [22]. Succinate dehydrogenase (SDH) is crucial as it intimately involved in both carbon metabolism and cellular respiration [23]. It is recognized as complex II within the mitochondrial respiratory chain and plays a pivotal role in the oxidation of succinate to fumarate, thereby fueling mitochondrial respiration [24,25]. As a result, altering the SDH is anticipated to decrease the cellular utilization of succinate, potentially leading to its accumulation, and to hinder the efficiency of cellular respiration [24]. Similarly, the compromised cellular respiration and ATP depletion occurred during APAP-induced hepatic injury [22,26,27]**.** And, there is no approved therapeutics against late stages or established paracetamol-induced liver injury, though different researchers reported mitochondria targeted different therapeutic approaches [3,28]. However, the mechanism by which the reactive metabolite NAPQI interacts with mitochondrial proteins or respiratory chain and subsequently formation of the NAPQI-protein adducts remains uncertain [29,30]. Hence, the aim of this research is to elucidate the intricate molecular mechanism underlying the interaction between NAPQI and mitochondrial complex II, specifically succinate dehydrogenase. This investigation employs a bioinformatic approach to delve into the implications of APAP toxicity. The holistic nature of this analysis aims to yield a significant understanding of the mechanistic pathway, potentially revealing prospective drug targets for countering APAP-induced liver injury.

## 2 Methods

### 2.1 Protein target and ligand preparation

The cytosolic and mitochondrial proteins were obtained from the RCSB PDB database (https://www.rcsb.org/). The downloaded structures were visualized and modified using the PyMOL Molecular Graphics System, version 2.5 Schrödinger, LLC [31]. Before the molecular docking experiment, all the heteroatom residues were cleaned. APAP (PubChem CID: 1983) and NAPQI (PubChem CID: 39763) are two ligands that were retrieved as Structure-Data file (SDF) 3D structures from the PubChem database (https://pubchem.ncbi.nlm.nih.gov/) [32,33].

### 2.2 Molecular docking performance and visualization

The docking calculations were performed using AutoDock Vina [34] and PyRx [35]. Each of the dockings was considered three times and mean and standard deviation were calculated. Redocking was performed by AutoDock Vina tool for the reliability of the software, cross-validation of binding affinity calculations, and consistency of the docking algorithm. The exhaustiveness was maintained as 20 exclusively for finding the best binding pose. For succinate dehydrogenase (PDB ID: 1ZOY) [36,37] grid box was positioned at the standard value (Center box: X = 89.3256, Y = 55.0384, Z = 106.18239), and the dimension of the box was set to X = 82.8283, Y = 81.2259, Z = 127.7991 Å. The results of docking were presented, and expressed as a negative value in units of kcal/mol, with lower scores indicating more favorable binding interaction [38]. Additionally, molecular graphics visualization of docking complexes was done by utilizing the BIOVIA Discovery Studio Visualizer (https://discover.3ds.com/discovery-studio-visualizer-download) [36].

### 2.3 Molecular dynamics simulations

100 ns molecular dynamic (MD) simulations were used to assess the binding stability and compactness of receptor and receptor-ligand complexes [39]. The thermodynamic stability of complexes was examined using the GROningen MAchine for Chemical Simulation (GROMACS) (version 2020.6). To mimic the inner mitochondrial membrane, complexes were integrated into a system comprised of 37 % phosphatidylcholine (POPC), 31 % phosphatidylethanolamine (POPE), 29 % cardiolipin (CL), phosphatidylinositol (PI), and TIP3 water model using CHARMM-GUI [40,41]. The K and CL ions were chosen for charge neutralization, and the CHARMM36 m force field was used to minimize system energy [39]. The equilibrium process was carried out by isothermal isochoric (NVT), and subsequently, isobaric (NPT) equilibration of the system was executed. The root mean square deviation (RMSD), root mean square fluctuation (RMSF), radius of gyration (Rog), solvent accessible surface area (SASA), and hydrogen bonding were used to assess the stability of the receptor-ligand complexes. After extracting the trajectory files, graphs were produced utilizing the Grace tool (https://plasma-gate.weizmann.ac.il/Grace/).

### 2.4 Protein-protein interaction network

To get protein-protein interaction data for this investigation, we used the STRING (Search Tool for Retrieval of Interaction Genes/Proteins) database (https://string-db.org/). STRING is a comprehensive online resource that integrates both experimental and predicted interactions among proteins [42]. The interaction analysis was carried out by selecting the highest confidence (0.900) and a maximum number of interactions was selected the 10 (ten). The investigation into protein-protein networking involved an in-depth analysis of three distinct processes; biological, molecular, and cellular processes, utilizing a web-based tool for Gene Ontology (GO) enrichment and pathway analysis (https://bioinformatics.com.cn/) [43].

### 2.5 Metabolic pathway analysis

The metabolic pathway of tricarboxylic acid (TCA) cycle and production of ROS induced by metallic compounds was analyzed using the Kyoto Encyclopedia of Genes and Genomes (KEGG) (https://www.genome.jp/kegg/pathway.html) database [44].

## 3 Results

### 3.1 Molecular docking of ligands and the SDH

Different mitochondrial and cytosolic proteins were reported to interact with NAPQI and at first, we carried out the binding affinity of those proteins with NAPQI using PyRx listed in Table 1. We observed that Glutamine synthase, Glutamate dehydrogenase, Thioether S-methyltransferase, glutathione S-transferase alpha-1, glutathione synthase I, succinate dehydrogenase, and electron transfer flavoprotein shown strong binding affinity (−6.0 kcal/mol or lower).

**Table 1:** Binding affinity prediction of mitochondrial and cytosolic proteins with NAPQI using PyRx.

Protein name	PDB ID	Binding affinity (kcal/mol)	Reference
Ribonuclease Binase	4HAA	−5.0 ± 0.158	[45]
Glutathione S-transferase alpha 1	3KTL	−6.27 ± 0.057	[26]
Glutathione S-transferase Mu 1	7OPY	−5.2 ± 0.152	
Glutathione S-transferase Mu 2	1HNA	−4.633 ± 0.048	
Glutathione S-transferase Pi	3GSS	−5.133 ± 0.104	
Succinate dehydrogenase	1ZOY	−6.7 ± 0.081	[46]
Electron transfer flavoprotein	1EFV	−6.3 ± 0.081	
Glutamine synthase	2QC8	−6.033 ± 0.046	[47]
Glutamate dehydrogenase	1L1F	−6.1 ± 0.00	
Aldehyde dehydrogenase	1ZUM	−5.9 ± 0.25	
N-10 formyltetrahydrofolate	4TT8	−5.966 ± 0.236	
Glutathione peroxidase	1GP1	−4.433 ± 0.044	
Thioether S-methyltransferase	2A14	−6.666 ± 0.044	
Bovine inorganic pyrophosphate	1FAJ	−5.133 ± 0.115	
Tropomysin 5	5JLF	−5.233 ± 0.190	
Selenium-binding protein	2JZ7	−5.466 ± 0.057	
Methionine adenosyl transferase	1O90	−5.933 ± 0.047	
Protein synthesis initiation factor 4A	1QDE	−4.966 ± 0.081	
ATP synthase alpha subunit	6J5I	−5.5 ± 0.2449	
Carbonic anhydrase III	1Z93	−5.233 ± 0.407	
Urate Oxidase	5LL1	−5.266 ± 0.057	
2,4-dienyl Co-A reductase	1PS9	−5.866 ± 0.057	
Glutathione reductase	3GRS	−5.67 ± 0.141	[48]
Mammalian thioredoxin reductase	3EAN	−5.833 ± 0.057	[49](Jan et al., 2014)
Glutathione synthase I	2HGS	−6.2 ± 0.00	[50]

As Succinate dehydrogenase (SDH) is responsible for cellular respiration, and SDH shown comparatively strong binding affinity (−6.7 ± 0.081 kcal/mol). Therefore, further molecular docking analyses were carried out to forecast the binding affinity and interaction residues between receptors and ligands using AutoDock Vina [34] and PyRx [35]. APAP shows the binding affinity −6.5 ± 0 kcal/mol and −5.8 ± 0.2 kcal/mol through PyRx and Auto Dock Vina respectively. Whereas, NAPQI exhibits more strong binding affinities of −6.7 ± 0.08 kcal/mol and −6.1 ± 0.2 kcal/mol by PyRx and Auto Dock Vina respectively. Table 2 provides a comprehensive overview of molecular interactions between the ligands and the targeted receptor, including their corresponding binding affinity and the interacting residues.

**Table 2:** Prediction of binding affinity and interaction profiling of ligands and the succinate dehydrogenase (SDH) using PyRx and AutoDock Vina.

Ligands	PyRx				AutoDock Vina			
	Binding affinity (kcal/mol)	Residue	Bond distance (Å)	Bond type	Binding affinity (kcal/mol)	Residue	Bond distance (Å)	Bond type
**APAP (CID-1983)**	−6.5 ± 0	Leu51	2.75	Conventional hydrogen	−5.8 ± 0.2	Leu51	2.74	Conventional hydrogen
		Lys50	2.28	Conventional hydrogen		Lys50	2.28	Conventional hydrogen
		Lys50	4.79	Pi-Alkyl				
		Ala179	2.55	Conventional hydrogen		Ala179	2.54	Conventional hydrogen
		Ala179	4.91	Pi-Alkyl				

**NAPQI (CID-39763)**	−6.7 ± 0.08	Thr214	2.93	Conventional hydrogen	−6.1 ± 0.2	Ala179	2.30	Conventional hydrogen
		Thr214	3.92	Pi-Sigma		Ala27	2.25	Conventional hydrogen
		Lys50	4.04	Pi-Alkyl		Gly26	2.36	Carbon hydrogen
		Ala179	2.09	Conventional hydrogen		Lys50	5.58	Pi -anion
						Phe178	4.613	Pi -anion

APAP: Paracetamol; NAPQI: N-acetyl-p-benzoquinonimine; CID: Compound ID.

**Fig. 1:**
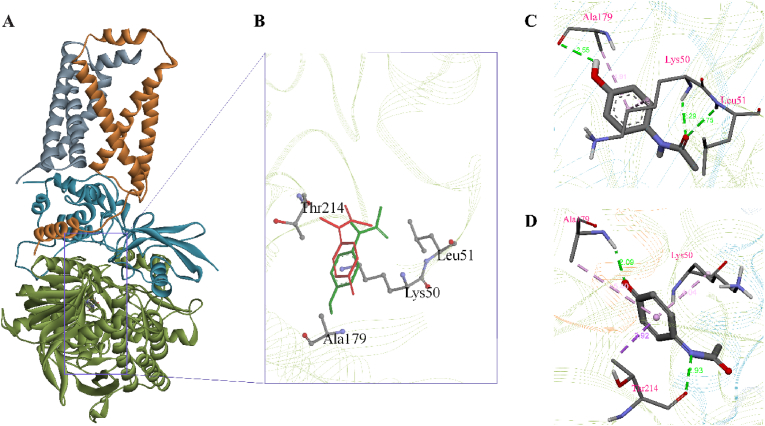
**The docking-based ligand interaction and their interacting residues**. A) Solid ribbon representation of succinate dehydrogenase (SDH), with SDH-A, SDH-B, SDH-C, and SDH-D subunits colored green, teal, orange, and gray, respectively. B) Residues in the SDH-A subunits associated with the two ligands. C) 3D interactions of APAP with succinate dehydrogenase. D) 3D interactions of NAPQI with succinate dehydrogenase. *Green, pink, and purple colors represent conventional hydrogen bond, pi-alkyl bond, and pi-sigma bonds respectively.*

APAP displays interactions involving three hydrogen bonds, as well as two pi-alkyl bonds (Fig. 1C). On the other hand, NAPQI exhibited two hydrogen bonds, along with two pi-alkyl bonds (Fig. 1D). In addition, NAPQI established a pi-sigma bond. Results from molecular docking studies suggest that NAPQI may bind with Complex II more efficiently compared to APAP.

### 3.2 Molecular dynamics simulation of SDH protein and its ligands APAP and NAPQI

Molecular dynamics (MD) simulations mimic cellular conditions, aiding in the assessment of protein-ligand complex stability and behavior. To better understand the conformational changes of the protein in the complex, a 100ns MD simulation of the protein in connection with the specific ligand was performed in this study.

#### 3.2.1 RMSD analysis of SDH-NAPQI and SDH-APAP complexes

**Fig. 2:**
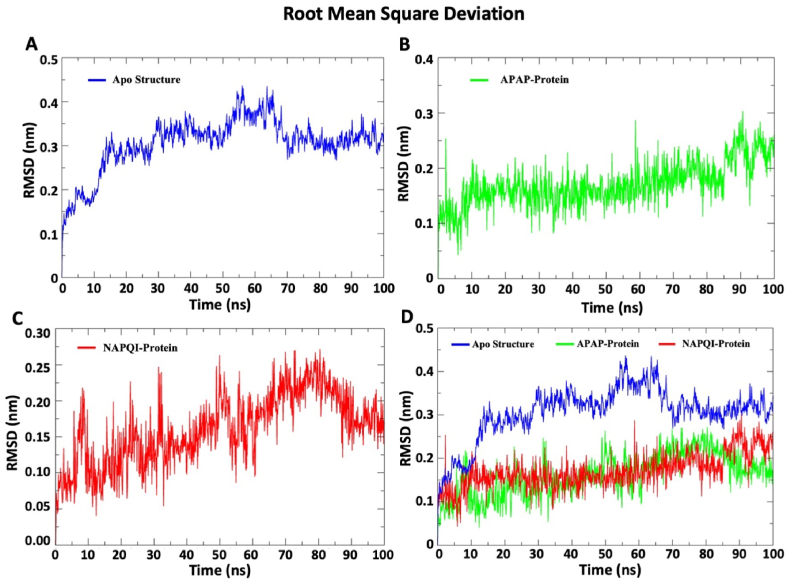
**The graphical representation of RMSD data. The colors bar represents three distinct setups of simulation; blue color for apo structure, green color for APAP-Protein complex, and red color for NAPQI-Protein complex.** (A) The RMSD of the backbone after isq fit to the backbone of protein only system. (B) The RMSD of protein in the presence of APAP was measured based on the backbone atoms of the complex system. (C) The RMSD of protein in the presence of NAPQI was measured based on the backbone atoms of the complex system. (D) This is the overlapping graph of the apo protein, APAP complex, and NAPQI complex for comparative analysis. This graphical representation offers insights into structural variations within the protein backbone under different conditions, facilitating a deeper understanding of conformational changes induced by apo structure, APAP, and NAPQI. *RMSD: Root mean square deviation; APAP: Paracetamol; NAPQI: N-acetyl-p-benzoquinonimine.*

The acceptable range of the root mean square deviation (RMSD) change within protein-ligand complexes is between 0.1 nm and 0.3 nm. Elevated RMSD values indicate substantial conformational changes within the protein-ligand complexes. The RMSD profiles has been demonstrated in Fig. 2. The apo structure RMSD values gradually increased and After 75 ns, the value is relatively stable. After an initial period of stability, the APAP and NAPQI complexes exhibited synchronous RMSD profiles, averaging around 0.2 nm. Overall, protein-ligand complex structure showed lower RMSD values than protein only structure all over the simulation timeframe.

#### 3.2.2 RMSF analysis of SDH-NAPQI and SDH-APAP complexes

**Fig. 3:**
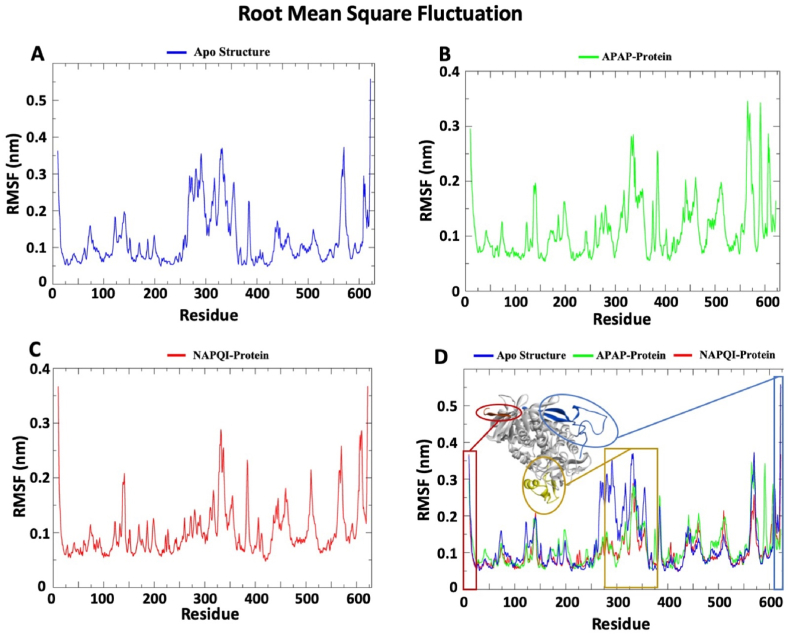
**The graphical representation of RMSF data of SDHA subunit. The colors bar represents three distinct setups of simulation; blue color for apo structure, green color for APAP-Protein complex, and red color for NAPQI-Protein complex.** (A) The RMSF of the backbone after isq fit to the backbone of protein only system. (B) The RMSF of protein in the presence of APAP was measured based on the backbone atoms of the complex system. (C) The RMSF of protein in the presence of NAPQI was measured based on the backbone atoms of the complex system. (D) This is the merged graph of the apo protein, APAP complex, and NAPQI complex for comparative analysis. Maximum fluctuation observed in the C-terminal region (colored blue), the N-terminal region (colored red) and middle residues (colored yellow). This graphical representation offers insights into structural variations within the protein backbone under different conditions, deepening our understanding of conformational changes induced by apo structure, APAP, and NAPQI. Apo structure exhibits wider flexibility in the middle compared to the other two complexes, indicating a more compact structure in the complexed states. *RMSF: Root mean square fluctuation; APAP: Paracetamol; NAPQI: N-acetyl-p-benzoquinonimine.*

Root Mean Square Fluctuation (RMSF) analysis was employed to discern the regional flexibility of the protein. Higher values correlate with increased flexibility at specific amino acid positions. The RMSF profiles (Fig. 3) of apo structure and two ligand compounds binding with SDHA subunits are shown. The apo structure showed the maximum flexibility in the C-terminal domain. Both complex showed higher fluctuation at N and C terminal. In general, protein looping-generating regions have higher flexibility than other secondary structures. The apo structure showed a considerably wider peak in the 250 to 350 amino acid position. Although the two complexes have similar trends in fluctuation, NAPQI showed lower fluctuations compared to APAP.

#### 3.2.3 SASA analysis of SDH-NAPQI and SDH-APAP complexes

**Fig. 4:**
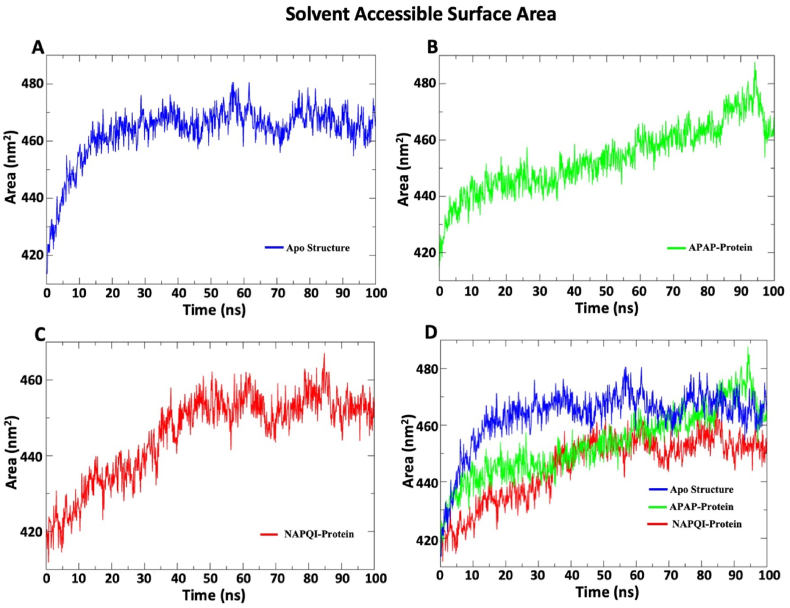
**The graphical representation of SASA values. The colors bar represents three distinct setups of simulation: blue color for apo structure, green color for APAP-Protein complex, and red color for NAPQI-Protein complex.** (A) The SASA values of protein only system. (B) The SASA of protein in the presence of APAP. (C) The SASA of protein in the presence of NAPQI. (D) In this overlapping graph, the apo protein, Protein-APAP complex, and Protein-NAPQI complex were analyzed comparatively. Apo protein exhibits higher exposure of water might indicate more open or flexible structure compared to complexed with APAP and NAPQI. This graphical representation provides insights into variations in the solvent-accessible surface area among different protein states, offering glimpse into the structural dynamics and interactions in the presence of APAP and NAPQI. *RMSF: Root mean square fluctuation; APAP: Paracetamol; NAPQI: N-acetyl-p-benzoquinonimine.*

Solvent Accessible Surface Area (SASA) analysis assists in forecasting the stability of a protein's hydrophobic core. The SASA values illustrate the interplay between protein stability and solvent accessibility. In Fig. 4, for apo structure the SASA value was gradually increased after 50 ns it was relatively stable. The protein complex with APAP displays a gradual increase in SASA values, leveling off at around 460–470 nm^2^ after 80 ns. In contrast, complexes with NAPQI exhibited progressive increase until 60 ns, therefore converging at an average value of ∼450 nm^2^. The noticeable reduction in protein backbone exposure to the solvent, particularly in response to NAPQI bindings compared to APAP bindings, indicates a relatively higher structural stability of the hydrophobic core. This suggests that the interaction with NAPQI may contribute to more compact and stable conformation of the protein, potentially influencing its overall functionality.

#### 3.2.4 Radius of gyration of SDH-NAPQI and SDH-APAP complexes

**Fig. 5:**
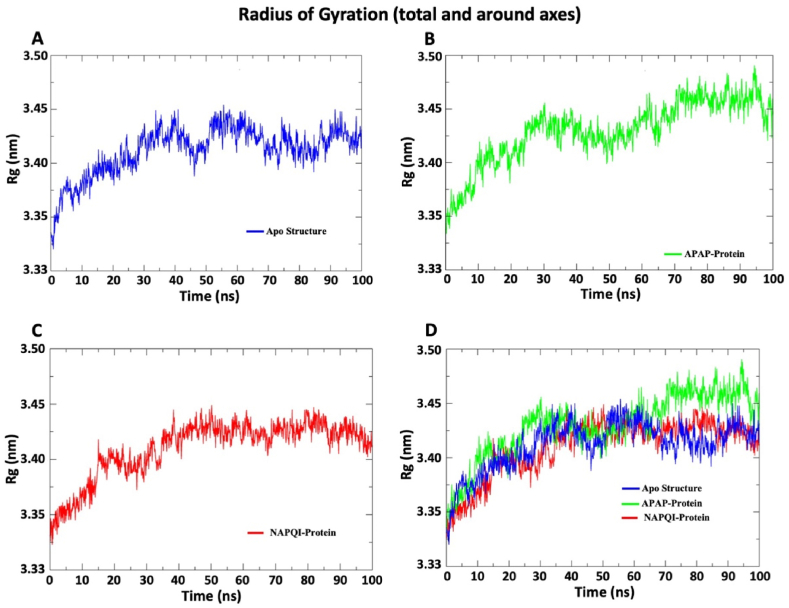
**The graphical representation of Rog values. The colors bar represents three distinct setups of simulation: blue color for apo structure, green color for APAP-Protein complex, and red color for NAPQI-Protein complex.** (A) The Rog values of protein-only system. (B) The Rog of protein in the presence of APAP. (C) The Rog of the protein in the presence of NAPQI. (D) In this graph, overlapping graph of the apo protein, APAP complex, and NAPQI complex for comparative analysis. APAP-protein complex exhibits a higher Rog peak at 60 ns, and maintaining elevated value until the end of 100 ns simulation, in contrast to other two structures. This graphical representation provides insights into variations in the Rog among different protein states, offering a glimpse into folding of the conformation and dynamic behavior. *RMSF: Root mean square fluctuation; APAP: Paracetamol; NAPQI: N-acetyl-p-benzoquinonimine.*

The radius of gyration (Rog) represents how spread out or concentrated the mass of an object is around its center. A relatively steady value of radius of gyration means stable folding of protein. Fluctuation of the radius of gyration implies the unfolding of the protein. Fig. 5 shows radius of gyration profile of three system, where protein has increasing Rog values during the simulation. For NAPQI-protein complex, first 40 ns Rog values were increasing after that graph of the complex was steady and compact. This steady and compact graph represent stable folding. Throughout the 100 ns simulation, NAPQI has lowest Rog values. The sustained lower Rog for NAPQI implies a more concentrated mass distribution around the center, reflecting a potentially more stable and compact conformation of the protein-ligand complex. The structural differences inferred from Rog values between APAP and NAPQI contribute valuable insights into their respective impact on the dynamic behavior and stability of the protein complex over the simulation time.

#### 3.2.5 Hydrogen bond profile of SDH-NAPQI and SDH-APAP complexes

**Fig. 6:**
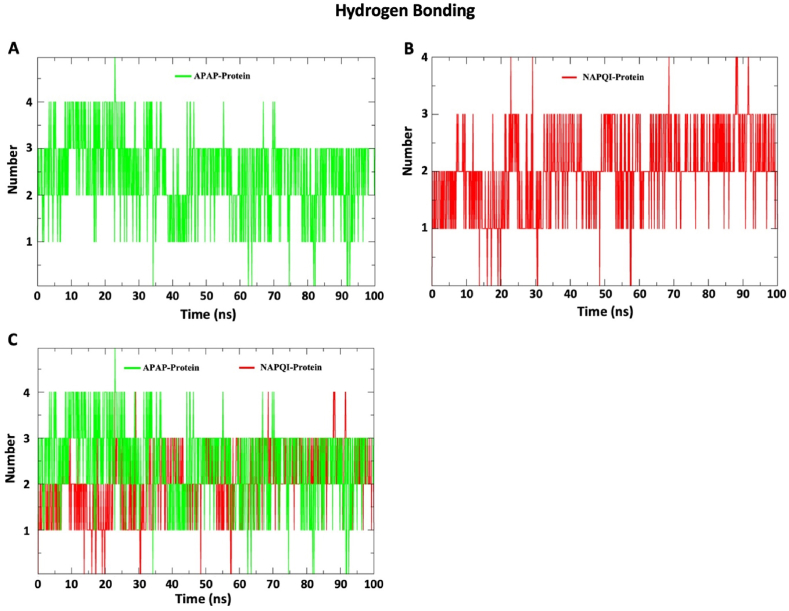
**The graphical representation of hydrogen bonding count of 100 ns simulation.** (A) Hydrogen bonding profile of APAP-Protein complex colored as green. (B) Hydrogen bonding profile of NAPQI-Protein complex colored as red. (C) In this graph, overlapping graph of the APAP complex, and NAPQI complex for comparative analysis. This graphical representation of hydrogen bonding provides insights into the dynamic interactions and structural relationships within these complexes, offering a detailed view of hydrogen bond form and persist over the course of the simulation. *APAP: Paracetamol; NAPQI: N-acetyl-p-benzoquinonimine.*

The hydrogen bond profiles of the two complexes are shown in Fig. 6. Hydrogen bonds between NAPQI and protein shown in the color red start the interaction with the 1–2 scale of hydrogen bond number. Ignoring the minor fluctuations, the number of hydrogen bonds remains on a 1–3 scale. On the other hand, the APAP profile shows a higher number of hydrogen bonds up to 25 ns. The APAP hydrogen bond number fluctuates from 0 to 4. APAP exhibited a maximum of 5 hydrogen bonds, observed near 25ns.

The study examined APAP and NAPQI's binding dynamics with SDH. Both complexes remained within the acceptable RMSD range (0.1–0.3 nm) and displayed distinct RMSF profiles. SASA analysis revealed differences in hydrophobic core stability, and Rog values indicated NAPQI's steady interaction with 1–2 bonds, contrasting with APAP's fluctuating bond count, which peaked at 25 ns. These results offer insights into the ligands' binding behavior with SDHA subunits.

### 3.3 Protein-protein interaction networks of succinate dehydrogenase flavoprotein subunit

**Fig. 7:**
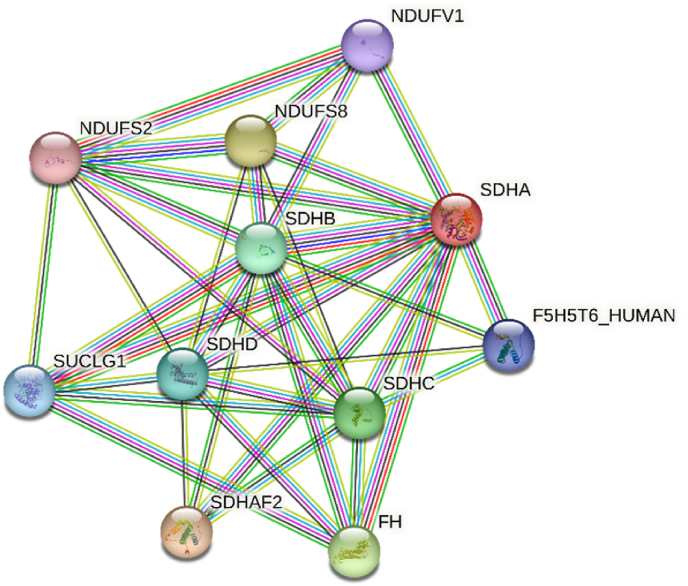
**Protein-protein interaction (PPI) networks of succinate dehydrogenase flavoprotein subunit.** In the visual representation, colored nodes with 3D structures symbolize all the protein proteins produced by a single protein-coding gene locus. The edges in the diagram depict protein-protein associations, highlighting meaningful connections. Interaction lines are color-coded, with cyan and pink representing confirmed interactions and green, red, and blue signifying predicted interactions. The interactions don't necessarily indicate physical binding between proteins. *SDHA: Succinate dehydrogenase flavoprotein subunit; SDHB: Succinate dehydrogenase iron-sulfur subunit; SDHC: Succinate dehydrogenase cytochrome b b560 subunit; SDHD: Succinate dehydrogenase cytochrome subunit; FH: Fumarate hydratase; SDHAF2: Succinate dehydrogenase assembly factor 2; SUCLG1: Succinate CoA ligase subunit alpha; NDUFS2: NADH dehydrogenase iron-sulfur protein 2; NDUFV1: NADH dehydrogenase flavoprotein 1; NDUFS8: NADH dehydrogenase iron-sulfur protein 8; F5H5T6_HUMAN: uncharacterized protein.*

In this study using STRING database, a comprehensive analysis of protein-protein interactions was conducted, revealing crucial interaction partners within the network (Fig. 7). These partners include essential components such as succinate dehydrogenase [ubiquinone] flavoprotein subunit (SDHA), succinate dehydrogenase [ubiquinone] iron-sulfur subunit (SDHB), succinate dehydrogenase cytochrome *b*560 subunit (SDHC), succinate dehydrogenase [Ubiquinone] cytochrome *b* small subunit (SDHD), fumarate hydratase (FH), succinate dehydrogenase assembly factor 2 (SDHAF2), succinate-CoA ligase [ADP/GDP-forming] subunit alpha (SUCLG1), NADH dehydrogenase [ubiquinone] iron-sulfur protein 2 (NDUFS2), NADH dehydrogenase [ubiquinone] flavoprotein 1 (NDUFV1), NADH dehydrogenase [ubiquinone] iron-sulfur protein 8 (NDUFS8), and uncharacterized protein (F5H5T6_HUMAN). The details of protein-protein interaction and enrichment were supported in Supplementary Fig. 1. These interactions shed light on critical cellular processes, including energy metabolism and mitochondrial function, offering valuable insights into the functional relationships among these proteins in our study.

### 3.4 Metabolic pathway analysis for succinate dehydrogenase

**Fig. 8:**
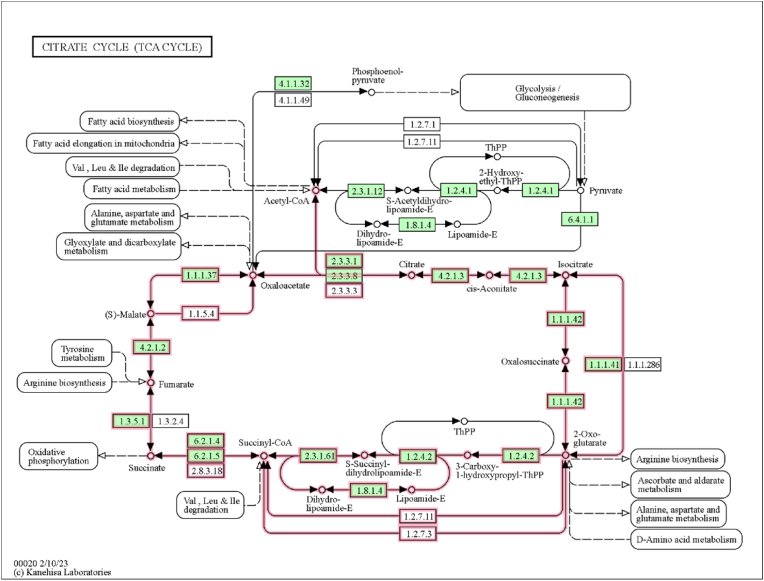
**Succinate to fumarate conversion in the TCA cycle**. This KEGG pathway diagram illustrates the conversion of succinate to fumarate within the TCA cycle. In this enzymatic reaction, succinate is oxidized to fumarate, accompanied by the transfer of electrons to the electron transport chain (ETC). Succinate is converted into fumarate by succinate dehydrogenase (1.3.5.1) is one of the critical conversions in the TCA cycle. The released electron is utilized in the oxidative phosphorylation process.

In this present study, we conducted metabolic pathway analysis, with a particular focus on the tricarboxylic acid (TCA) cycle. During this analysis, a crucial enzyme with the KEGG identifier 1.3.5.1, which corresponds to the flavoprotein subunit of succinate dehydrogenase was identified (Fig. 8). Succinate dehydrogenase plays a critical role in this metabolic pathway by catalyzing the conversion of succinate to fumarate while simultaneously transferring electrons to the electron transport chain, thereby contributing to the production of adenosine triphosphate (ATP) in cellular respiration [51]. Reactive oxygen species (ROS) are commonly produced in the mitochondrial matrix as a natural byproduct of the electron transport chain during cellular respiration. This KEGG analysis is further linked with supporting mitochondrial RO production in the presence of metals (Supplementary Fig. 2).

## 4 Discussion

In this computational study, we predicted the molecular interaction of paracetamol-derived reactive metabolite NAPQI with mitochondrial complex II succinate dehydrogenase through conventional hydrogen, Pi-Alkyl, and Pi-Sigma bonds. This molecular interaction is also confirmed by molecular dynamics analysis through the stability and compactness of the SDH-NAPQI complex. Our study observation confirms the previous experimental findings that SDH is very sensitive to NAPQI and the succinate-driven compromised respiration subsequently generates APAP-inducing irreversible hepatic injury in mice [26]. It was also reported that NAPQI binds to mitochondrial protein targets during paracetamol toxicity, causing a reduction in energy production, generation of reactive oxygen species, and cellular death [52]. When it comes to lowering mitochondrial respiratory capacity, maximal respiratory rates, and ATP generation, NAPQI is significantly more effective than APAP [53].

Succinate dehydrogenase is a multi-subunit enzyme in the TCA cycle and a subunit (SDHA) of it involves the oxidation of succinate to fumarate [54,55]. Electrons are then used to decrease Flavin adenine dinucleotide (FAD) to FADH_2_, which in turn reduces ubiquinone to ubiquinol in the respiratory chain. Through a series of reduction steps, the electron is transported from succinate to ubiquinol in this forward process. The reduced ubiquinone pool provides electrons for the reversal process. The enzyme succinate dehydrogenase is capable of producing ROS both in the forward and reverse directions. The competitive binding of dicarboxylates in the substrate binding site of complex II shows that the fully reduced, vacant flavin site is the major source of oxygen radical production [56].

Molecular docking study showed NAPQI has a slightly higher affinity for SDHA compared to APAP. Analysis of the APAP and NAPQI binding sites indicated that both ligands partially occupy the FAD binding sites (Supplementary Fig. 3). The cofactor-bound succinate dehydrogenase crystal structure revealed the establishment of hydrogen bonds at the sites LysA50, GlyA65, AlaA179, AsnA413, SerA414, and LeuA415 between the main chain atoms of FAD and SDHA, which support earlier studies [37]. The higher binding potential of NAPQI suggests that it is more predisposed to binding succinate dehydrogenase (SDHA) within mitochondrial complex II. This observation is crucial in understanding the molecular mechanisms underlying acetaminophen-induced liver injury, particularly in elucidating how NAPQI interacts with mitochondrial enzymes like SDHA. In addition, Electron transfer flavoprotein (ETF), a mitochondrial heterodimeric protein act as an electron acceptor from several mitochondrial dehydrogenases [57,58] also shown significant binding affinity (−6.3 ± 0.081 kcal/mol) with NAPQI. This ETF further transfer the electrons through ETF-ubiquinone oxidoreductase to the main electron transport chain [57]. Our ongoing research focuses on an in-depth analysis of this Electron Transfer Flavoprotein in this context.

The findings of the RMSD analysis also revealed that in a few timeframes, the RMSD for APAP and NAPQI marginally overlapped. After attaching APAP and NAPQI to the SDHA chain, the protein backbone fluctuated, indicating the N and C terminals had the largest variations. This finding implies that the binding properties of APAP and NAPQI are comparable. NAPQI's gyroscope radius and SASA value are smaller than APAP's, despite this. A molecule is more stable when its SASA value is lower because it is less exposed to environmental variables like solvent molecules or chemical reactions [59]. Additionally, the hydrogen bond profile indicates that NAPQI interacts in a distinct way with protein residues via hydrogen bonds. Overall, the molecular dynamics simulation of this work demonstrated that NAPQI favorably interacts with succinate dehydrogenase and may have the ability to change the enzymatic activity of this membrane-integrated protein SDH. By illuminating the stability and binding procedures of protein-ligand complexes, this prediction could aid in the investigation of the interactions between proteins and ligands.

NAPQI selectively inhibits mitochondrial complex II and reduces the rates of ATP biosynthesis driven by succinate [22]. Protein-protein interaction networks reveal important associations related to mitochondrial energy metabolism. Succinate dehydrogenase and fumarate dehydrogenase are ubiquitously expressed and play a vital role in ATP production through the mitochondrial respiratory chain [60]. SDHA2 directly interacts with SDHA and is required for FAD insertion [61]. However, the compromised activity of complex II after binding of NAPQI in the flavin site could potentially reduce energy production. On the other hand, the oxidative stress followed by elevated ROS in mitochondria by environmental xenobiotics such as arsenic [62], chromium [63], lead [64], and mercury [65] leads to the continuous release of superoxide. Mitochondrial membrane complexes are responsible for ROS generation as well as energy production. Therefore, the overall impact of toxic drug metabolite NAPQI or chemical carcinogens on nucleophilic sites potentiates the cellular destruction events subsequently.

Previously it was reported that succinate dehydrogenase is a therapeutic target for bleomycin-induced Idiopathic pulmonary fibrosis [66], therefore this study provides a significant drug target SDH for APAP-induce hepatotoxicity. Considering the biological significance of mitochondrial membrane complex II in APAP toxicity, this computational study suggests that NAPQI has favorable binding in the flavin site of Succinate dehydrogenase, although further validation of this site towards an inhibitory enzymatic mechanism will be needed.

## 5 Conclusion

The interaction between NAPQI and mitochondrial succinate dehydrogenase (SDHA) poses a significant threat to cellular well-being, resulting in diminished energy production and the formation of detrimental reactive oxygen species. This investigation underscores SDHA's potential as a focal point for drug discovery and emphasizes the importance of comprehending the mechanistic pathways associated with complex II inhibition. Molecular dynamics simulations indicate that NAPQI binding to SDHA can impact enzymatic activity, highlighting the imperative for further exploration in this domain. In essence, this knowledge illuminates the intricate interplay between toxic metabolites, mitochondrial function, and their implications for cellular survival.

## Funding

There were no grants provided for this research by funding organizations in the public, private, or nonprofit sectors.

## Availability of data and material

The data underlying this article is available in the article and in its online supplementary material.

## CRediT authorship contribution statement

**Md Sahadot Hossen:** Data curation, Formal analysis, Investigation, Methodology, Validation, Visualization, Writing – original draft, Writing – review & editing. **Adiba Akter:** Data curation, Investigation, Methodology, Visualization, Writing – original draft. **Mahir Azmal:** Data curation, Formal analysis, Investigation. **Mostakim Rayhan:** Investigation, Validation. **Kazi Saiful Islam:** Writing – review & editing. **Md Mahmodul Islam:** Writing – review & editing. **Shamim Ahmed:** Supervision, Writing – review & editing. **Mohammad Abdullah-Al-Shoeb:** Conceptualization, Data curation, Methodology, Project administration, Supervision, Writing – original draft, Writing – review & editing.

## Declaration of competing interest

The authors declare that they have no known competing interest and they have taken all necessary precautions to preserve the intellectual property connected to this work and there are no barriers to publication that are related to intellectual property, including when to publish.

## Data Availability

Data will be made available on request.
