# Supplementary material for: Unveiling the molecular basis of paracetamol-induced hepatotoxicity: Interaction of N-acetyl-p-benzoquinone imine with mitochondrial succinate dehydrogenase
Source: Biochem Biophys Rep. 2024 May 7;38:101727. doi: 10.1016/j.bbrep.2024.101727 (PMC11098724; doi:10.1016/j.bbrep.2024.101727)
Supplement: Multimedia component 1 [file mmc1.docx]

**Supplementary Figures:**

#
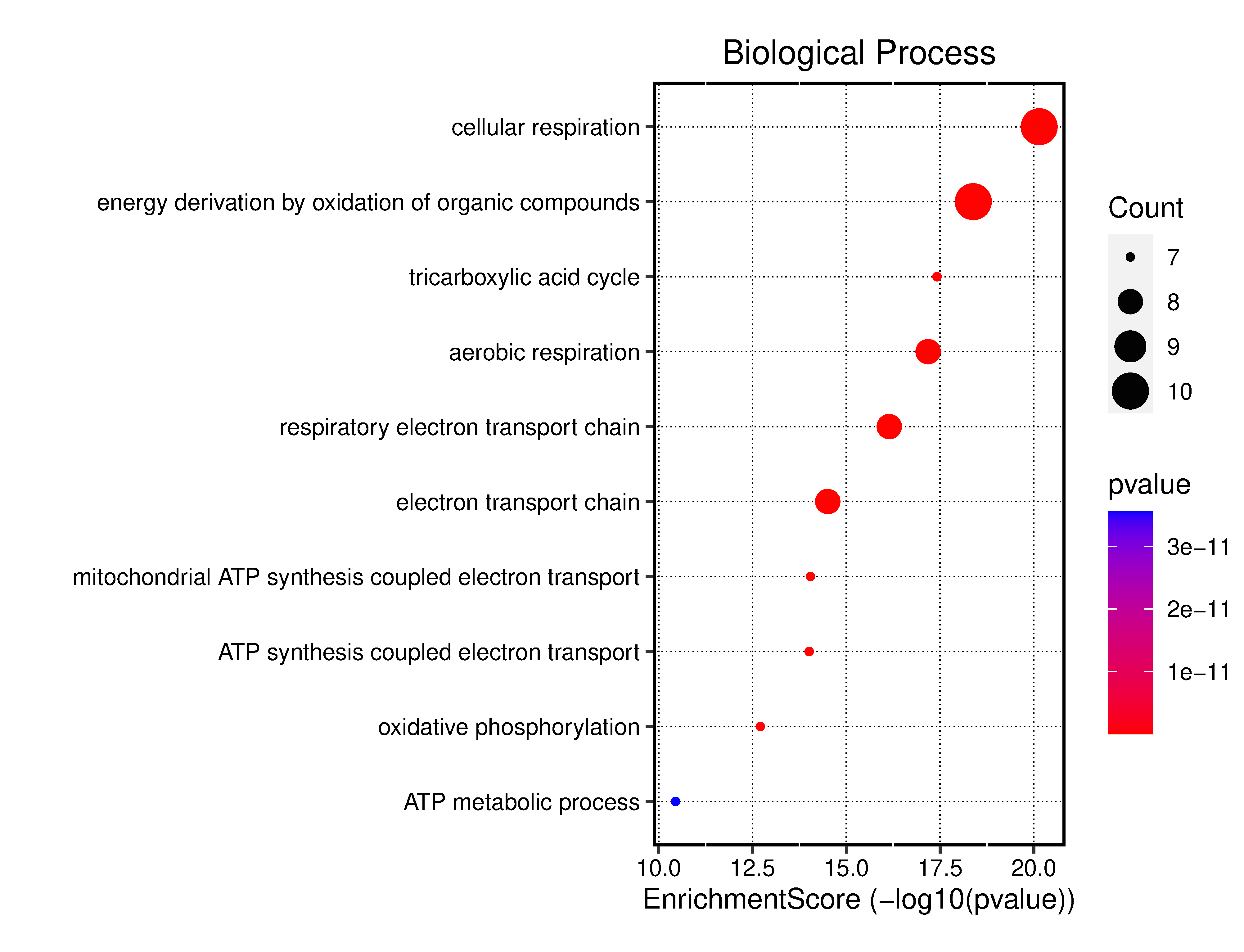


**Supplementary Figure 1.** The dot plot shows the biological process of succinate dehydrogenase (SDH) subunits and its associated proteins in the GO ontology. *The dot size on right side represents the counts of associated proteins. The enrichment score is given as the negative logarithm of the p-value where lower p-value means higher biological enrichment.*

#
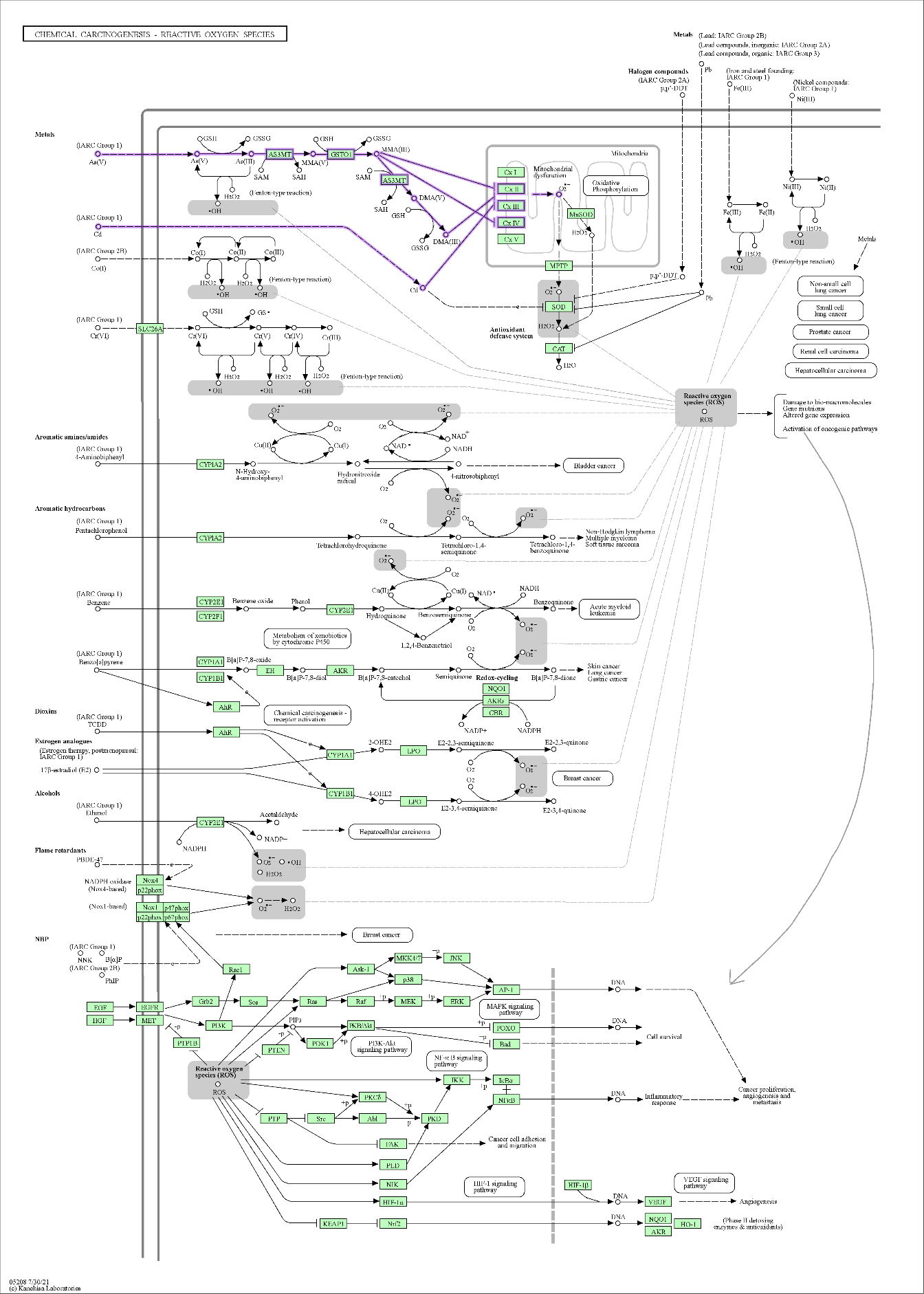


**Supplementary Figure 2.** The role of metallic compounds in reactive oxygen species (ROS) generation. *The Arsenic (v) accept the electrons from GSH and convert in Arsenic (III). The Arsenic (III) promotes a group of enzymes which utilizes SAM and GSH as substrate. The product MMA DMA from this sequential reaction pathway may directly block the Complex II activity cause ROS formation in mitochondria.*


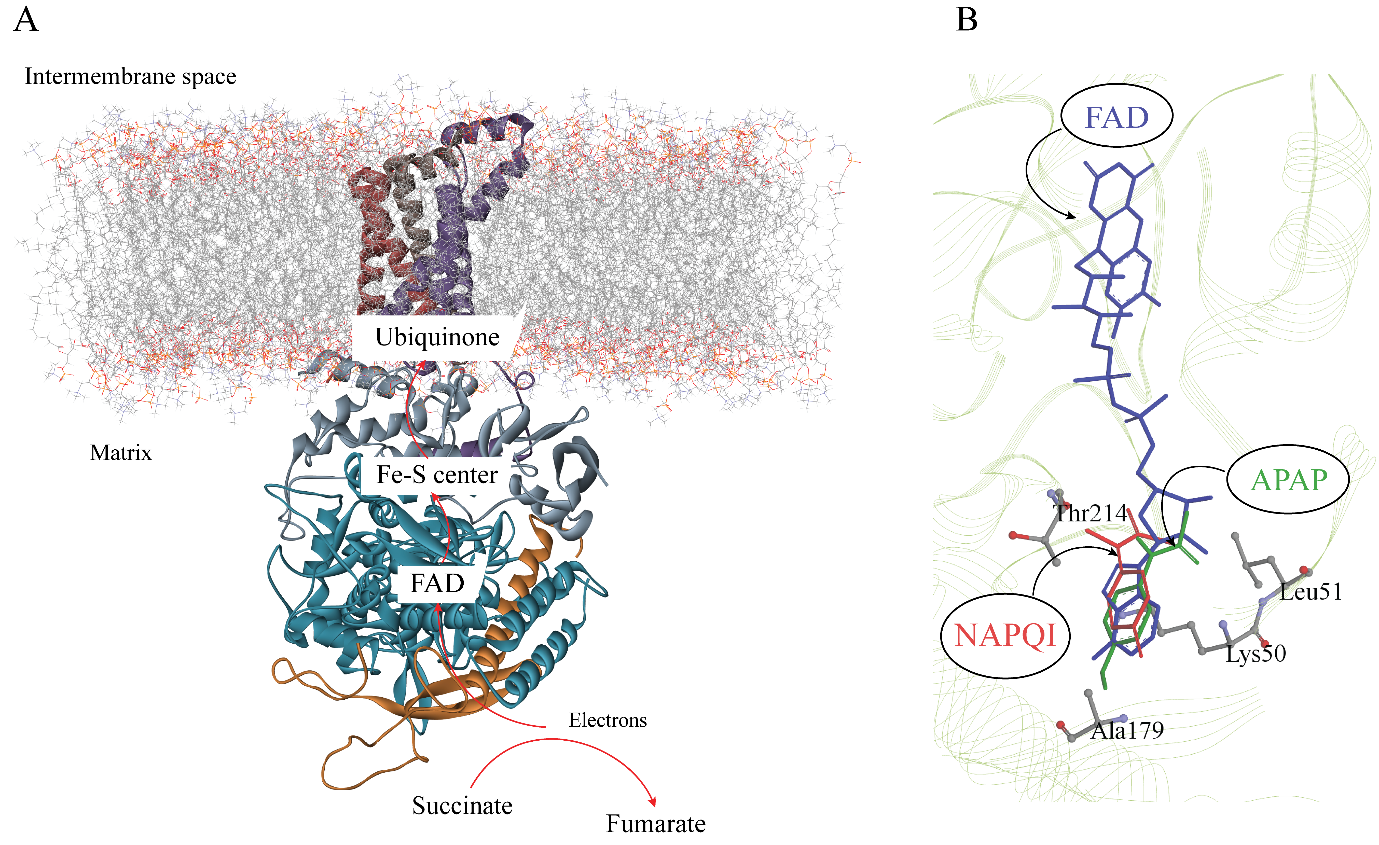


**Supplementary Figure 3.** The figure represents electrons transferring mechanism in mitochondria and amino acid specific binding of ligands. *A) Succinate dehydrogenase (SDH) integrated in mitochondrial membrane where involved in electron transfer from succinate to ubiquinone through flavin adenine dinucleotide (FAD) site to Fe-S cluster to ubiquinone. B) FAD binding site is partially occupied by APAP and NAPQI. Few residues in the FAD site is common binding residues for FAD, APAP, and NAPQI.*
